# Etiology and Antimicrobial Susceptibility of Pathogens Associated with Urinary Tract Infections among Women Attending Antenatal Care in Four South African Tertiary-Level Facilities, 2015–2019

**DOI:** 10.3390/antibiotics10060669

**Published:** 2021-06-04

**Authors:** Thembekile Zwane, Liliwe Shuping, Olga Perovic

**Affiliations:** 1 Faculty of Health Sciences, School of Public Health, University of the Witwatersrand, Private Bag 3 Wits, Johannesburg 2050, South Africa; 2 Centre for Healthcare-Associated Infections, Antimicrobial Resistance and Mycoses, National Institute for Communicable Diseases, Division of the National Health Laboratory Service, Private Bag X4 Sandringham, Johannesburg 2131, South Africa; LiliweS@nicd.ac.za; 3 South African Field Epidemiology Training Program, National Institute for Communicable Diseases, Division of the National Health Laboratory Service, Private Bag X4 Sandringham, Johannesburg 2131, South Africa; 4 Department of Clinical Microbiology and Infectious Disease, Faculty of Health Sciences, School of Pathology, University of the Witwatersrand, Private Bag 3 Wits, Johannesburg 2050, South Africa

**Keywords:** community-acquired urinary tract infection, antimicrobial susceptibility, multidrug-resistant uropathogens

## Abstract

In South Africa, uncomplicated community-acquired UTIs (CA-UTIs) are treated empirically; however, the extent of antibiotic resistance among these pathogens is not well known. We conducted a descriptive cross-sectional study of women attending ANCs at four tertiary public-sector hospitals in Gauteng. Female patients aged 15–49 years, with urine cultures performed between January 2015 and December 2019, were included. A case of culture-confirmed UTI was defined as any woman with ≤2 uropathogens with a bacterial count of ≥105 colony-forming units per ml for at least one pathogen. We identified 3558 cases of culture-confirmed UTIs in women with a median age of 30 years (interquartile range; 25–35). *E. coli* accounted for most infections (56% (1994/3558)), followed by *E. faecalis,* with a prevalence of 17% (609/3558). The prevalence of *K. pneumoniae* was 5% (193/3558), 5% (186/3558) for *S. agalactiae,* and 5% (179/3558) for *P. mirabilis*. Ninety-five percent (1827/1927) of the *E. coli* and 99% of the *E. faecalis* (301/305) isolates were susceptible to nitrofurantoin. Common uropathogens showed high susceptibility to first-line antibiotics, gentamicin and nitrofurantoin, as recommended for use in primary healthcare settings. Overall, our study provided an indication of the level of antimicrobial resistance in the four facilities.

## 1. Introduction

Antimicrobial resistance (AMR) is a growing global public health concern as it threatens the effective control and treatment of bacterial infections [1]. AMR patterns are evolving at an alarming rate due to the overuse of empiric broad-spectrum antimicrobials, and urinary tract infections (UTIs) are increasingly caused by multidrug-resistant (MDR) pathogens, particularly in healthcare facilities [2,3]. Antibiotics are commonly prescribed to treat uncomplicated UTIs in primary and secondary healthcare settings, and as a result, antibiotics used to treat UTIs have also changed over the years due to increasing AMR levels [2,4,5]. UTIs account for 25% of all clinical bacterial infections, and the European Survey of Antibiotic Consumption reported that infections with MDR bacteria result in about 25,000 deaths annually among Europeans [4,6,7]. 

Community-acquired UTIs (CA-UTIs) are the second most common infection in primary care [8]. Compared to other women of reproductive age, pregnant women acquire UTIs more frequently due to anatomical and, to a lesser extent, immunological changes associated with pregnancy [9]. Some of the factors that contribute to the development of UTIs during pregnancy include ureteral dilation, along with decreased ureteral tone due to hormonal effects and pressure from the growing uterus [10,11]. These factors further contribute to increased urinary stasis, which may encourage the selective growth of bacteria in the urine [11]. CA-UTIs affect between 28% and 30% of pregnant women worldwide [9,12]. In Sub-Saharan Africa, including South Africa, there are few research studies on CA-UTIs among pregnant and non-pregnant women [13,14,15]. Moreover, AMR in CA-UTIs in South Africa has not been thoroughly documented. Two single-center studies conducted among hospitalized pregnant women in South Africa reported a UTI prevalence of 5% in Durban and 8.3% in Cape Town [12,16]. However, in these studies, AMR patterns were not reported. 

To determine the etiologic agents and their susceptibility profiles among patients with UTIs, urine culture is the method of choice [16]. However, culture is not routinely used in primary care, and information on circulating pathogens and susceptibility profiles is often used to guide empiric therapy [17]. According to the recently updated South African Standard Treatment Guidelines and Essential Medicines List of 2020, gentamicin is recommended as the first-line treatment of uncomplicated UTIs in adults [18]. However, gentamicin is contra-indicated in pregnancy, therefore either fosfomycin or nitrofurantoin is recommended as the first-line treatment of UTIs for pregnant women [18]. Ciprofloxacin is recommended for all adults with complicated UTIs in South Africa [18]. 

In resource-limited settings, syndromic treatment approaches are introduced for uncomplicated system organ infectious diseases, however, continuous monitoring of etiology and antimicrobial susceptibility testing of bacterial pathogens is required to prevent the development of antimicrobial resistance. We aimed to determine the etiology and susceptibility patterns of uropathogens circulating in the community, analyzing pregnant women attending antenatal clinics (ANCs) in Gauteng with culture-confirmed UTIs. We anticipated that our study findings would provide information to guide the empiric treatment of all women with UTIs in primary healthcare.

## 2. Results

**Figure 1:**
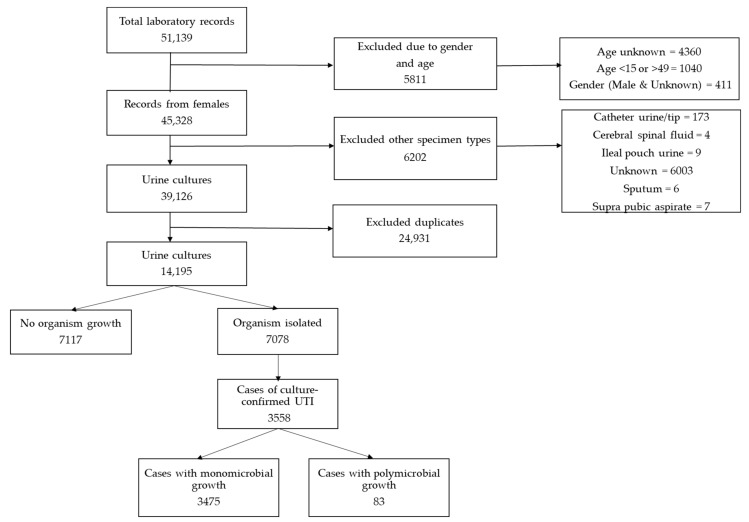
The number of culture-confirmed cases of urinary tract infections (UTIs) among women, with urine specimens collected while attending antenatal care at the four tertiary hospitals in Gauteng, 2015–2019.

A total of 51,139 laboratory records were retrieved over the five-year period; 36,944 (63%) were excluded based on age, sex, other specimen types and duplication, and the remaining 14,195 urine cultures were submitted from 13,955 women. The positivity rate was 50% (7078/14,195) and the contamination rate was 25% (3520/14,195). Among all positive urine cultures, 50% (3558/7078) represented cases of culture-confirmed UTIs, and 2% (83/3558) of these were polymicrobial infections (two uropathogens) (Figure 1). Most cases were obtained at CHBAH (46% (1651/3558)) and CMJAH (32%, (1142/3558)). Of the remaining cases, 12% (430/3558) were from RMMCH, and 9% (335/3558) from SBAH. The median age of the patients was 30 years (interquartile range, 25–35) and the majority of the cases were aged 25–34 years (52% (1860/3558)). 

**Figure 2:**
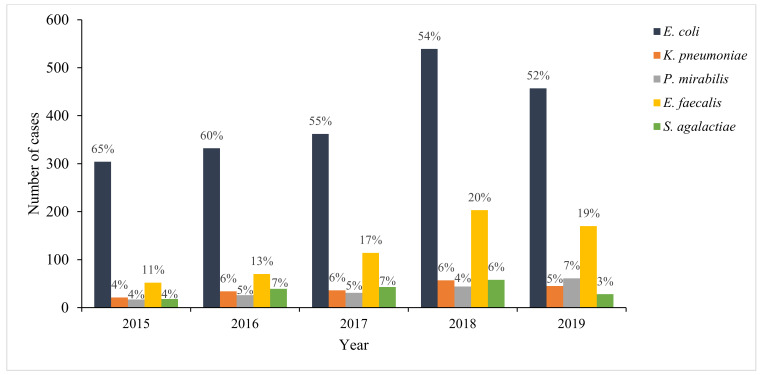
Annual distribution of predominant uropathogens among cases of culture-confirmed urinary tract infection in antenatal clinics at four tertiary public-sector hospitals in Gauteng Province, 2015–2019. Number of cases were: *n* = 471 (2015); *n* = 554 (2016); *n* = 655 (2017); *n* = 996 (2018); *n* = 882 (2019).

Among the cases of culture-confirmed UTIs, the most common pathogens were *E. coli* (56% (1994/3558)) and *E. faecalis* (17% (609/3558)) (Figure 1). The prevalence of *S. agalactiae* was 5% (186/3558), with 5% (179/3 558) for *P. mirabilis* and 5% (193/3558) for *K. pneumoniae*. Other Gram-negative uropathogens accounted for 8% (298/3558) of the infections, and these included, but were not limited to, *Klebsiella* spp. (3% (111/3558)), *Enterobacter* spp. (2% (67/3558)), and *Acinetobacter* spp. (0.4% (14/3558)). The prevalence of other Gram-positive uropathogens was 3% (99/3558), and the most predominant species among these were *Staphylococcus* spp. (2% (86/3558)) and *Enterococcus* spp. (0.3% [10/3558]). *Candida* spp. accounted for 0.2% (6/3558) of the uropathogens. *E. coli* and *E. faecalis* remained the most dominant uropathogens throughout the analysis period (Figure 2).

Susceptibility testing of the predominant uropathogens is shown in Table 1. Among culture-confirmed cases, 82% (95% confidence interval (CI) 81–84 (1594/1936)) of the *E. coli* isolates were susceptible to amoxicillin/clavulanic acid and 88% (95% CI 86–88 (1626/1852)) were susceptible to ciprofloxacin. Ninety-five percent (95% CI: 94–96 (1827/1927)) of the *E. coli* isolates were susceptible to nitrofurantoin and 93% (95% CI 92–94 (1835/1970)) to gentamicin. *E. coli* isolates were less susceptible to ampicillin/amoxicillin (24%, 95% CI 22–26 (470/1947)) and trimethoprim/sulfamethoxazole (38%, 95% CI 36–40 (742/1942)). *E. coli* demonstrated reduced susceptibility to cefuroxime (86%, 95% CI 84–88 (904/1050)) and cefazolin (88%, 95% CI 86–90 (1122/1275)), while susceptibility to cefotaxime/ceftriaxone was 93% (95% CI 91–94 (1805/1948)) and 95% (95% CI 93–95 (1880/1988)) for cefepime. The majority of *K. pneumoniae* isolates were susceptible to ciprofloxacin (96%, 95% CI 91–98 (172/180)), gentamicin (95% CI 90–97 (178/188)) and trimethoprim/sulfamethoxazole (75%, 95% CI 68–81 (151/202)). Forty percent (95% CI 34–48 (80/198)) of the *K. pneumoniae* isolates were susceptible to nitrofurantoin. Among the *K. pneumoniae* isolates, 89% (95% CI 83–92 (165/186)) were susceptible to cefuroxime, 91% (95% CI 86–95 (177/194)) to cefotaxime/ceftriaxone, 92% (95% CI 87–95 (185/201)) to cefepime and 97% (95% CI 84–100 (31/32)) to cefazolin.

Of the 176 *P. mirabilis* isolates tested, 98% (95% CI 94–99) and 99% (95% CI 96–100) were susceptible to amoxicillin/clavulanic acid and ciprofloxacin, respectively, and only 51% (95% CI 43–58) were susceptible to ampicillin/amoxicillin. *E. faecalis* showed high susceptibility to ampicillin/amoxicillin (99%, 95% CI 99–100 (612/613)) and nitrofurantoin (99%, 95% CI 97–100 (301/305)). Thirty-seven percent (95% CI 24–51 (20/54)) of the *E. faecalis* isolates were susceptible to trimethoprim/sulfamethoxazole. 

Overall, 4% (155/3558) of the cases were infections with MDR uropathogens (resistant to ciprofloxacin, ampicillin and trimethoprim/sulfamethoxazole), 87% of which (*n* = 134) were *E. coli*.

## 3. Discussion

In our study, 88% of culture-confirmed UTI infections among pregnant women in antenatal care were due to five uropathogens, of which *E. coli* and *E. faecalis* were the most common. With the exception of trimethoprim/sulfamethoxazole, susceptibility to antimicrobials was generally high, with some variation by pathogen. Although susceptibility to nitrofurantoin was low for *K. pneumoniae* isolates, it was high for *E. coli* and *E. faecalis*. Ciprofloxacin and gentamicin susceptibility was high overall, and the prevalence of MDR uropathogens was low. 

As is consistent with a number of other studies in patients with CA-UTIs, *E. coli* was the most prevalent uropathogen among cases in our study [14,19]. The 56% prevalence of *E. coli* in our study was lower than the 80% previously reported in a similar study performed in Gauteng, but was similar to a study done in KwaZulu Natal (54%) [14,20]. The possible reasons for the differences with the study in Gauteng could be due to the study design and the population under survey. The previous study enrolled 200 symptomatic non-pregnant women, who may differ from pregnant women with respect to the causative pathogens. *E. faecalis* was the second most prevalent uropathogen in our study, with a prevalence of 17%. This was in contrast to previous community-based South African studies, one including all women, and one including only pregnant women. In these studies, *E. faecalis* accounted for 4% of the UTIs, despite being the most common Gram-positive uropathogen [20,21,22]. Pregnancy usually results in physical changes to the genital tract, and these changes may increase the risk of colonization and infection with Gram-positive and other uncommon pathogens [9,20]. For example, in other studies of pregnant women, *Candida* spp. were the predominant pathogens [20,23]. 

Overall, there was high susceptibility to those antibiotics recommended for first-line treatment in primary care settings (i.e., nitrofurantoin and gentamicin), although this differed by the common pathogen. In a laboratory-based study between 2015 and 2017 at CMJAH, susceptibility to ciprofloxacin and nitrofurantoin was 71% and 70%, respectively [22]. This lower susceptibility compared to our study was most likely due to the relatively higher resistance rates among pathogens isolated from hospitalized patients [24]. *E. coli* was susceptible to nitrofurantoin (96%) and, to a lesser extent, to ciprofloxacin (88%). Although the proportion of *E. coli* isolates that were susceptible to ciprofloxacin was high, resistance among *E. coli* isolates was above the 10% threshold set by the Infectious Diseases Society of America (IDSA) for treatment modification [25]. Above the 10% resistance threshold, the IDSA recommends that alternative antimicrobials be used for the empirical treatment of CA-UTIs [25]. 

Although the overall susceptibility to nitrofurantoin was high in our study, this was an exception with *K. pneumoniae* (40%) and *P. mirabilis* isolates. Regarding *P. mirabilis* isolates, we need to emphasize that *Proteus* spp. are intrinsically resistant to nitrofurantoin, and it should not be considered as a treatment option [26]. We examined the susceptibility to third- and fourth-generation cephalosporins (i.e., cefotaxime and cefepime) which are used as an indication for extended-spectrum beta-lactamase (ESBL) production, and are therefore resistant to this class of antibiotics. The overall prevalence of ESBL-producing strains in *E. coli* was between 6–7% and 8–9% in *K. pneumoniae*. This finding was consistent with studies reporting increasing ESBL production among strains isolated from urine [27]. The prevalence of ESBLs differs between community-acquired and healthcare-associated infections in South Africa. A higher prevalence of ESBLs has been reported in hospitalized patients (8–13%) compared to patients with community-acquired infections (0.3–5%) [24,28,29]. Another single-center study in hospitalized patients and outpatients reported an ESBL prevalence of 23% [22]. These differences highlight the importance of periodic assessment of antimicrobial susceptibility patterns in both community and healthcare settings, as resistance rates differ depending on the setting [30]. 

In our study, the prevalence of MDR uropathogens was low, as we described a specific population and included a first episode of UTI. Fosfomycin and nitrofurantoin are suitable for treating UTIs with MDR uropathogens, and nitrofurantoin is more suitable for pregnant women [31]. We need to emphasize that there are no breakpoints for certain Gram-positive organisms, non-enterobacterales and enterobacterales other than *E coli* for reporting nitrofurantoin according to the guidelines of both the CLSI and European Committee on Antimicrobial Susceptibility Testing (EUCAST), which means it should not be recommended to clinicians. We did not have data on fosfomycin, as susceptibility testing was not routinely performed for this antibiotic in the NHLS laboratories. However, resistance to nitrofurantoin was low in our study, suggesting that it remains a suitable first-line treatment for CA-UTIs and for the treatment of MDR infections. Compared to MDR pathogens isolated from patients in the healthcare setting, community-acquired pathogens have no antibiotic resistance phenotype, and they are more stable in the absence of antibiotic pressure normally found in healthcare settings [22,32]. Therefore, the emergence of MDR pathogens in the community should be avoided through responsible use of antibiotics, with more directed treatment.

The findings of our study should be interpreted in the context of the study limitations. First, our study included only pregnant women attending antenatal care, thus findings may not be generalizable to other population groups. In addition, we limited our analysis to Gauteng, and our results may not represent resistance patterns in other parts of South Africa. However, the strength of our study was that it included women attending antenatal clinics at large public-sector facilities that offer services to individuals from all parts of the province, and thus was likely to be representative of antimicrobial susceptibility patterns of the pathogens circulating in communities in Gauteng Province.

## 4. Materials and Methods

### 4.1. Study Design

This was a descriptive cross-sectional study including women attending antenatal clinics (ANC) with culture-confirmed UTIs, in four tertiary-level facilities in Gauteng; Charlotte Maxeke Johannesburg Academic Hospital (CMJAH), Rahima Moosa Mother and Child Hospital (RMMCH), Steve Biko Academic Hospital (SBAH) and Chris Hani Baragwanath Academic Hospital (CHBAH). We conducted secondary data analysis using laboratory data from the National Health Laboratory Service Corporate Data Warehouse (NHLS CDW), which stores all laboratory results of urine cultures performed in public-sector facilities. We obtained urine culture results from 2015 to 2019 and used laboratory-defined ANC test codes to identify urine specimens from patients seen at antenatal clinics at the four facilities. Limited patient information, including dates of birth and sex, and laboratory results, including bacterial colony-forming unit counts, organisms cultured, and antimicrobial susceptibility testing (AST) results were retrieved. Standard operative procedures for urine cultures and AST (automated breakpoints—Vitek 2®bioMerieux, disk diffusion methods or others) were used at the NHLS laboratories, and interpretation AST results were performed according to the Clinical and Laboratory Standards Institute (CLSI) guidelines throughout the study period [33].

### 4.2. Definitions

A case of culture-confirmed UTI was defined as any woman of childbearing age (15–49 years) with a positive urine culture consisting of ≤2 uropathogens with an organism growth of ≥100,000 colony-forming units per ml for at least one of the isolated organisms. We used the Centers for Disease Control and Prevention National Healthcare Safety Network (CDC NHSN) guidelines to distinguish uropathogens from contaminants among positive cultures [34]. 

MDR uropathogens were defined as organisms resistant to three or more classes of antimicrobials [35], including beta-lactam antibiotics (penicillin derivatives, carbapenems and cephalosporins), fluoroquinolones, aminoglycosides, folate pathway inhibitors (i.e., trimethoprim-sulfamethoxazole) and nitrofurantoin.

### 4.3. Exclusion Criteria

Additional urine cultures from the same patient that met the case definition and were collected within six months of the first urine culture were regarded as part of the initial UTI episode and were excluded. Male patients, women patients aged <15 years and >49 years, and those with non-urine specimen types were excluded. Patients with missing information on study inclusion criteria, such as date of birth or age, sex, specimen type and collection date, and organism name, were also excluded.

### 4.4. Data Analysis

The positivity and contamination rates were calculated. We determined the urine positivity rate by dividing the number of urine cultures where any organism was isolated by the total number of urine cultures performed. We determined the contamination rate by dividing the number of urine cultures that yielded a non-uropathogen by the total number of urine cultures. For each antimicrobial, susceptibility was determined by dividing the number of antimicrobial susceptible uropathogens by the total number of uropathogens with AST results.

## 5. Conclusions

This study provides updated profiles of infectious causes of UTIs and antimicrobial susceptibility, and may be used to guide empirical UTI treatment for female outpatients in Gauteng. Additional studies including other population groups, such as non-pregnant women, patients seeking care in private facilities, and patients in other regions of the country, could be considered for better representation. In conclusion, there is a need for regular community-based surveillance of antimicrobial resistance patterns for empirical treatment recommendations at the primary healthcare level.

## Figures and Tables

**Table 1:** Antimicrobial susceptibility of predominant uropathogens among cases of culture-confirmed urinary tract infection in antenatal clinics at four tertiary public-sector hospitals in Gauteng Province, 2015–2019.

Antibiotic	*E. coli*	*K. pneumoniae*	*P. mirabilis*	*E. faecalis*	*S. agalactiae*	All
	n * (% [95% CI])						
Ciprofloxacin	1626 (88% (86–89))	172 (96% (91–98))	157 (99% (96–100))	243 (96% (92–98))	16 (100% (79–100))	2483 (90% (89–91))
Ampicillin/amoxicillin	470 (24% (22–26))	0 (0% (0–1))	89 (51% (43–58))	612 (99% (99–100))	63 (100% (94–100))	1215 (38% (37–40))
Trimethoprim/sulfamethoxazole	742 (38% (36–40))	151 (75% (68–81))	85 (49% (41–56))	20 (37% (24–51))	16 (94% (71–100))	1234 (35% (33–36))
Nitrofurantoin	1827 (95% (94–96))	80 (40% (34–48))	-	301 (99% (97–100))	-	2374 (83% (82–85))
Amoxicillin/clavulanic acid	1594 (82% (81–84))	169 (85% (80–90))	165 (98% (94–99))	-	-	2034 (81% (79–82))
Gentamicin	1835 (93% (92–94))	178 (95% (90–97))	170 (94% (90–97))	-	-	2482 (93% (92–94))
Cefepime	1880 (95% (93–95))	185 (92% (87–95))	180 (99% (97–100))	-	-	2514 (94% (93–95))
Cefotaxime/ceftriaxone	1805 (93% (91–94))	177 (91% (86–95))	177 (99% (96–100))	-	84 (100% (96–100))	2489 (93% (92–94))
Cefuroxime	904 (86% (84–88))	165 (89% (83–92))	103 (99% (95–100))	-	-	1225 (85% (83–87))
Cefazolin	1122 (88% (86–90))	31 (97% (84–100))	86 (99% (94–100))	-	-	1367 (86% (84–88))

* Number of susceptible isolates, 95% CI = 95% confidence interval.

## Data Availability

Data supporting the results of this study are not publicly available but can be made available on request.
